# Detection of dengue in German tourists returning from Ibiza, Spain, related to an autochthonous outbreak, August to October 2022

**DOI:** 10.2807/1560-7917.ES.2024.29.14.2300296

**Published:** 2024-04-04

**Authors:** Lucía García-San-Miguel, Jaume Giménez-Durán, Gabriela Saravia-Campelli, María Cruz Calvo-Reyes, Beatriz Fernández-Martínez, Christina Frank, Hendrik Wilking, Ramón García Janer, Miguel Ángel Miranda, Esteban Aznar Cano, Mª José Sierra Moros, Antonio Nicolau Riutort

**Affiliations:** 1 Centro de Coordinación de Alertas y Emergencias Sanitarias, Ministerio de Sanidad, Madrid, Spain; 2 Conselleria de Salut i Consum, Illes Balears, Dirección General de Salud Pública y Participación, Palma de Mallorca, Spain; 3 Servicio de Epidemiología, Palma de Mallorca, Spain; 4 IDISBA, Institut d’Investigació Sanitària Illes Balears, Palma de Mallorca, Spain; 5 Centro Nacional de Epidemiología, Instituto de Salud Carlos III, Madrid, Spain; 6 CIBER de Enfermedades infecciosas CIBERINFEC Network; 7 Department for Infectious Disease Epidemiology, Robert Koch Institute, Berlin, Germany; 8 Servicio de Salud Ambiental, Negociado zoonosis, Palma de Mallorca, Spain; 9 Applied Zoology and Animal Conservation research group, University of the Balearic Islands, Palma de Mallorca, Spain

**Keywords:** autochthonous dengue, outbreak, tourist, Aedes albopictus

## Abstract

In February 2023, German public health authorities reported two dengue cases (one confirmed, one probable) and four possible cases who travelled to Ibiza, Spain, in late summer/autumn 2022; the infection was probably acquired through mosquito bites. Case 1 visited Ibiza over 1 week in late August with two familial companions; all three developed symptoms the day after returning home. Only Case 1 was tested; dengue virus (DENV) infection was confirmed by presence of NS1 antigen and IgM antibodies. Case 2 travelled to Ibiza with two familial companions for 1 week in early October, and stayed in the same town as Case 1. Case 2 showed symptoms on the day of return, and the familial companions 1 day before and 3 days after return; Case 2 tested positive for DENV IgM. The most probable source case had symptom onset in mid-August, and travelled to a dengue-endemic country prior to a stay in the same municipality of Ibiza for 20 days, until the end of August. Dengue diagnosis was probable based on positive DENV IgM. *Aedes albopictus*, a competent vector for dengue, has been present in Ibiza since 2014. This is the first report of a local dengue transmission event on Ibiza.

**Key public health message**

**What did you want to address in this study and why?**

Dengue is a mosquito-borne influenza-like disease. *Aedes* mosquitos capable of transmitting dengue virus typically live in tropical areas. However, over the past 2 decades, *Aedes albopictus* has been establishing itself in several areas in central and southern Europe. Given virus introduction by travellers returning from dengue-endemic areas, transmission in Europe is possible. We describe the first local dengue outbreak on Ibiza, in the Balearic Islands, Spain.

**What have we learnt from this study?**

Our investigation provides an example of how a traveller returning from an endemic area and infected with dengue virus can start a local outbreak in Europe. The detection of dengue in persons living or travelling to non-endemic areas with the presence of *Ae. albopictus* mosquitoes must be rapidly communicated to public health authorities in order to guide relevant prevention and control measures to avoid further transmission.

**What are the implications of your findings for public health?**

In areas where *Ae. albopictus* is well established, travellers from dengue, Zika or chikungunya- endemic regions should protect themselves against mosquito bites, especially if they start feeling unwell. It is important that health authorities carry out communication campaigns to the local population to promote this recommendation.

## Background

Dengue is a vector-borne disease, endemic in most tropical countries. The World Health Organization (WHO) considers dengue as the most important viral disease transmitted by mosquitoes, particularly given the fact that its incidence has multiplied in recent decades and because of the rapid expansion of the disease to areas that were previously unaffected. Dengue is caused by the dengue virus (DENV), an RNA virus of the *Flaviviridae* family, and commonly transmitted to humans mainly through the bite of infected *Aedes* mosquitoes; other routes including sexual transmission are also possible [1,2]. The infection is asymptomatic in 40–80% of cases. When symptoms do occur, the clinical course is mild and self-limiting in most cases, usually presenting as an influenza-like syndrome with fever, muscle and joint pain, rash and fatigue; a small proportion of cases (< 5%) may develop severe dengue [1,3]. There is no specific treatment, but since 2021, a vaccine has been approved for use in endemic areas under certain circumstances.

The DENV perpetuates itself in a human-mosquito-human cycle in urban areas. When the female mosquito bites a viraemic individual, the virus enters its midgut and the extrinsic incubation period (EIP) begins, which lasts an average of 8–10 days, although it can vary depending on the ambient temperature. This period ends when the virus reaches the mosquito's salivary glands and the mosquito becomes infective. The infected person can transmit the virus to the vector while they are in the viraemic phase, which usually begins 1–2 days before the onset of fever and lasts between 4 and 7 days, with a maximum of 12 days [3,4]. 

In Europe, the last major dengue epidemic, vectored by *Aedes aegypti*, was reported in Greece in 1928 [5]. Subsequently, no cases caused by autochthonous vector transmission were detected until 2010. Since then, and up to the end of 2023, there have been sporadic cases of dengue and autochthonous vector-borne dengue outbreaks, generally small (1–13 cases, except in the 2022 and 2023 seasons in France, with 65 and 45 cases respectively, and Italy with 82 cases), in areas with the presence of *Ae. albopictus* in Croatia (2010), France (2010, 2013–15 and 2018–23), Spain (2018, 2019, 2022 and 2023) and Italy (2020 and 2023) [6]. In 2012, there was an outbreak of dengue, facilitated by presence of the vector *Ae. aegypti* on the island of Madeira (Portugal), during which more than 2,000 cases were detected. In total, until the end of 2023, 42 dengue outbreaks have been recorded in Europe [6,7]. The cumulative number of dengue cases in Europe by December 2023 was 275 [6].

In Germany and Spain, dengue remains a travel-associated disease. The only autochthonous cases reported in Germany to date have been rare nosocomial transmissions, although the competent vector *Ae. albopictus* has been present in parts of Germany since 2007 [8], mainly in the south-west area of the country [9]. In Spain, the first autochthonous dengue cases were identified in 2018: an outbreak of five related cases in the Region of Murcia and another unrelated case in Catalonia [2]. In 2019, two autochthonous cases were detected, one in Catalonia, and one in Madrid (likely sexual transmission [10]). Additionally, in 2023, another three autochthonous cases were detected in Catalonia [6]. According to the epidemiological surveillance data from the Balearic Islands, 44 travel-associated dengue cases (all residents of Spain) were notified from 2015 to 2023. Of these 44, seven were residents (permanent or temporary) of Ibiza; two of which were notified in 2022. The distribution of travel-associated dengue cases in the Balearic Islands is presented in Supplementary Figure S1. 

In Spain, *Ae. albopictus* was detected for the first time in Catalonia in 2004 [11]. According to the Spanish entomological surveillance data from 2009 to 2021, *Ae. albopictus* is established throughout the Mediterranean coast and in the Balearic Islands [12]. A map of the results from the vector surveillance is provided in Supplementary Figure S2. On Ibiza, *Ae. albopictus* was detected for the first time in 2014 in the town of Sant Antoni de Portmany through a citizen’s report and collection of adult mosquitoes [13]. In subsequent years, *Ae. albopictus* was detected in the five municipalities on the island. Between 2020 and 2021, more than 18,000 mosquitoes, including 10 different species collected from urban, peri-urban, rural and natural areas of the Balearic Islands, have been screened for different pathogens, including arboviruses [14]. A total of 844 *Ae. albopictus* females were negative for alphavirus and flavivirus, however, none of these were collected from Ibiza [14].

## Outbreak detection

On 1 February 2023, the German national public health institute, Robert Koch Institute (RKI) informed the Spanish National Centre for Epidemiology (NCE) of the Instituto de Salud Carlos III about the detection of two probable cases of dengue. The case patients had travelled separately to the Balearic Islands in August and October 2022, and had been notified to RKI as dengue cases on 13 September and 24 October 2022, respectively. Both cases had a positive DENV IgM test and reported Ibiza as the likely place of exposure. Since Spain is not regarded as an endemic country for dengue virus, additional laboratory confirmation was requested by RKI after receiving the notification. Confirmation and reporting of this confirmation was delayed until February 2023, when Germany could establish the relationship between the two cases and immediately informed Spain. 

Here, we report the epidemiological data of all related cases, the results of the entomological investigation and the control measures taken on Ibiza and at national coordination level. 

## Methods

### Case finding and epidemiological surveillance

Cases were detected through epidemiological surveillance at the national and local levels. 

In Germany, dengue has been notifiable for laboratories (and cases of haemorrhagic disease additionally by physicians) since 2001. According to the ‘Protection against Infection’ law, local health departments receive the notification, collect additional data, e.g. exposure, and send the pseudonymised case data to the RKI, where the national database is hosted. 

Epidemiological data from the German tourists were obtained by public health authorities in Germany and communicated by email and through the Early Warning and Response System of the European Union (EWRS) platform in a selective exchange between Germany and Spain. 

In Spain, dengue has been notifiable to the Spanish National Surveillance System (RENAVE) since 2015. For this outbreak, a description of historical travel-associated dengue was obtained both by the National Centre of Epidemiology (NCE) and the Epidemiology Service of the General Directorate of Public Health and Participation, Ministry of Health and Consumption of the Government of the Balearic Islands (Epi-Bal) records of nationally notifiable diseases to RENAVE. 

### Case definition 

In Spain, confirmed and probable case definitions for this outbreak were according to the European Union (EU) case definition [15]. Germany uses a slightly different case definition, but the classification of the German probable and confirmed cases is identical to the EU case definition. The confirmed case definition requires symptoms (at least fever) and laboratory confirmation. With exposure in dengue-endemic areas, even a single positive anti-DENV IgM result is accepted, but without such exposure, the more specific laboratory tests (NS1 antigen tests, PCR or serological confirmation by neutralisation assay) are required. The definition of a possible case for this study is a case fulfilling the EU clinical criteria, having an epidemiological link, but not fulfilling laboratory criteria.

### Environmental investigation

Data from *Ae. albopictus* establishment in Spain were obtained from entomological surveys conducted annually in all regions in Spain [12,15]. Technical reports on vector control were provided by local environmental public health authorities in the Balearic Islands and were also included in the description.

Climatic parameters as rain and average temperatures were obtained from the Ministry of Agriculture, Fisheries and Food [16].

### Calculation of transmission parameters

To calculate the mosquito infectivity period, we calculated the time lapse between the mosquito’s bite of a viraemic person to the end of the EIP (7 days), and the end of the median lifespan of mosquito according to the average temperature in Ibiza in August and October (7 weeks after EIP) [17,18]. The medium viraemia period considered was 2 days before and 7 days after symptom onset.

## Results

### Case description

The eventually confirmed case (Case 1) travelled from Germany to Ibiza for a week in late August 2022. They stayed at a private accommodation with two familial companions. The case developed symptoms compatible with dengue (fever, joint pain and rash) on the day after return to Germany. The diagnosis of dengue was obtained 8 days after return, with positive IgM and negative IgG serology. The case met the laboratory definition for a probable case, but since Spain is not a known endemic area, it overall did not meet the EU definition of even a probable case and thus Spain was not informed at that time. At the same time Case 1 developed symptoms, one travel companion had fever and runny nose, while another experienced sore throat, runny nose and joint pain. Neither required healthcare assistance and therefore diagnostic tests were not performed.

The eventual probable case (Case 2) travelled from Germany to Ibiza, to the same town as Case 1 and their company, for a week in early October, together with two familial travel companions and three additional groups of friends. On the day after returning to Germany, Case 2 developed symptoms: fever, headache, muscle and joint pain, retro-orbital pain and rash. A serological diagnosis of dengue was made 5 days after their return in Germany, based on a positive result for IgM detection without further determinations. At the time, this case alone also did not yet meet the probable status in the EU case definition. Both familial travel companions also presented mild symptoms compatible with dengue 1 day before and 3 days after Case 2 developed symptoms, but diagnostic tests were not performed. The family of Case 2 reported getting many mosquito bites in their accommodation room. None of the other travel companions noticed any symptoms.

RKI collected detailed information about Case 1 and 2 in January 2023 and could relate both cases epidemiologically (time and space). Only this epidemiological linkage changed their status to probable. Further laboratory tests were performed on samples from Case 1 and a positive DENV-NS-1 antigen test was obtained. In February 2023, Germany considered this case confirmed and informed Spain through EWRS. 

### Epidemiological investigation

In February 2023, upon receiving notifications about the two dengue cases from RKI, the NCE immediately informed the Alerts and Emergencies Coordinating Centre (CCAES) of the Spanish Ministry of Health. Thereafter, CCAES contacted the Epidemiology Service of the General Directorate of Public Health and Participation, Ministry of Health and Consumption of the Government of the Balearic Islands (Epi-Bal). To determine any related cases, the NCE and Epi-Bal began an investigation and identified two travel-associated cases of dengue in 2022, detected in Ibiza. 

Of these two travel-associated cases, the first case, who started the viraemia period in February 2022, was ruled out as the source case, as they had not visited the same places as the German cases; moreover, the period of viraemia was not during the vector season (Apr–Nov). The second travel-associated case, with permanent residence in a city on the Spanish mainland, visited a dengue-endemic country for 2 weeks at the end of July/beginning of August, and thereafter travelled to Ibiza. This case stayed for 20 days in the same town as the German cases until the end of August. On the day after leaving the dengue-endemic country and arriving on Ibiza, the case developed symptoms, including fever, severe headache, muscle and joint pain, nausea and vomiting. The diagnosis of dengue was reached at the beginning of September, given positive results for DENV IgM detection. According to the Spanish [19] and EU case definition [15], this case was considered a probable case.

### Environmental investigation

Upon detection of the travel-related case, the public health authorities of the Balearic Islands carried out an environmental epidemiological assessment. According to the patient’s statement, during the entire symptomatic (first 7 days of stay on Ibiza) and viraemic period (first 9 days on Ibiza), the case remained at their accommodation on the island, only leaving the premises to visit the healthcare centre located in Ibiza town. The residence was a recently built single-family home. The case stated that they did not observe the presence of mosquitoes at the house, nor noticed any mosquito bites. No information about sexual contact was collected. 

To assess the risk of transmission, the technicians from the environmental authority evaluated the environmental conditions, obtaining climatological data of maximum, average and minimum temperature, the external and internal environment of the house and the activities carried out by the case. The area where the house was situated consisted mostly of single-family homes and apartment complexes, with very few public areas. There were no scuppers, drains and grates on the streets, which are the predominant breeding sites of the *Aedes* mosquito.

Taking into account that the presumed source case stated that they had adopted individual protection measures and did not notice any mosquito bites, the evaluation concluded, assessing the risk for dengue as moderate. Therefore, the environmental public health authorities decided that the use of in situ adulticide treatments was not necessary. Recommendations to the municipality aimed to reinforce entomological surveillance, eliminate potential mosquito breeding sources and inform the public.

### Risk assessment

According to the average temperature recorded in Ibiza between August and October (27.25 °C August, 24.10 °C September, 19.91 °C October [20]) adults of *Ae. albopictus* were able to be active and transmit the virus, i.e. host-seeking and feeding behaviour.

The spatial coincidence of cases, the timing of the mosquito infectivity period after the EIP and the maximum lifespan of the mosquito suggest that the transmission from the source case to Case 1 could be through mosquito bites. The transmission to Case 2 through a mosquito bite was also plausible from a mosquito infected either from Case 1 or source case. Also, the possibility that transmission to other cases also occurred and went undetected cannot be ruled out. The timeline/temporal relationship of source and autochthonous cases is shown in the Figure.

**Figure:**
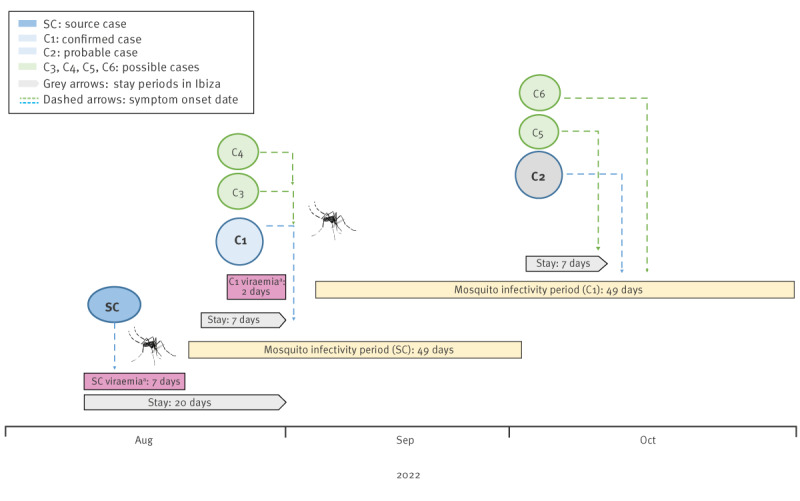
Temporal relationship of the source and autochthonous dengue cases during their stay in the same locality on the island of Ibiza, Spain, August–October 2022

^a^ Days of viraemia spent in Ibiza.

Source: Health Alerts and Emergencies Coordination Centre, Ministry of Health, Spain.

## Outbreak control measures

Control measures were executed at both the local and national levels in Spain. At the local level, after being alerted in February 2023 about the detection of autochthonous cases in Ibiza, the authorities of the Balearic Islands scheduled the reinforcement of entomological activities before the start of *Ae. albopictus* season in the first half of March 2023. The entomological measures were directed at the affected municipality where entomological surveillance and vector control, i.e. at breeding sites and adult foci where appropriate, were reinforced. Moreover, and above all, information to the public was promoted to encourage individual protective measures against mosquito bites. 

At the beginning of the 2023 season (1 May) and until the end of November, the regional authorities of the Balearic Islands (epidemiology and environmental areas), in coordination with different other actors, implemented the following measures (Box):

Box **Control measures implemented to prevent autochthonous dengue transmission, Ibiza, Spain, May–November 2023**

- **Blood donor screening:** In order to control transmission and detect asymptomatic cases, the Blood and Tissue Bank, in coordination with the National Centre for Microbiology, screened the blood from donors on the island of Ibiza. From 26 April to 19 December 2023, 1,300 blood samples from donors were screened using the In Vitro Qualitative Test for Direct Detection of Chikungunya Virus RNA (CHIKV) and Dengue Virus Serotypes 1-4 RNA (DENV) in Human Plasma (cobas CHIKV/DENV (Roche)). All samples yielded a negative result.
- **Enhanced surveillance:** A special surveillance for dengue (VEDENGUE) was launched on the island of Ibiza. According to the protocol, all suspected cases must be tested for dengue (PCR test) at the reference laboratory of the Balearic Islands, i.e. every person with regular residence on the island of Ibiza, regardless of whether or not they have recently visited dengue-endemic areas, and persons who meet the following clinical criteria: sudden fever onset, in the absence of another source of infection, and at least two of the following symptoms: severe headache, retro-orbital pain, muscle and joint pain, low back pain, nausea, vomiting or exanthema. From 1 May to 30 November 2023, the special dengue surveillance in Ibiza collected data from about 20 patients who were not detected as cases on any occasion.
- **Environmental action plan**: An environmental action plan was initiated, which was especially focused on the municipality where the outbreak was detected. This plan included reinforced entomological surveillance, control of breeding sources, chemical control of adult mosquitoes and information^a^ for citizens and tourist establishments to protect themselves from mosquito bites.
- **Citizen science:** The use of citizen science has been promoted through app platforms, i.e. Mosquito Alert. The use of these tools may help authorities communicate with citizens and vice versa, sharing information about bites, mosquito breeding sites and health promotion materials.

^a^ The island of Ibiza together with the neighbour island, Formentera, have been considered as one geographical unit, given the constant movement of people and vehicles between the two islands. Therefore, documentation on the *Aedes albopictus* (tiger mosquito) has been made available to all municipalities to have homogeneous information for the two islands.

At the national level, the CCAES coordinated the alert, promoting communication between the parties and participating in meetings with local authorities. From the CCAES, together with the National Centre of Epidemiology and National Microbiology Centre and the authorities of the Balearic Islands, a risk assessment was carried out at the national level and was published on the website of the Ministry of Health on 28 February 2023 [21]. Likewise, on the same day, timely communication to the European Centre for Disease Prevention and Control (ECDC), the European Union/European Economic Area (EU/EEA) countries and the World Health Organization (WHO) was sent through the EWRS platform and Epipulse, the European surveillance portal for infectious diseases. Before the start of the 2023 season, another meeting between CCAES and Balearic Public Health authorities was convened, which included technicians from the Environmental Health Service, the Blood and Tissue Bank and the Department of Agriculture. In Germany, in 2023, no further cases of dengue potentially infected in Spain were notified.

## Discussion

We highlight challenges of detecting dengue cases, both in the autochthonous population and in travellers to European countries that are non-endemic but where the vector is present. This fact is of utmost importance in order to prompt public health control measures. 

The environment in Spain meets the necessary conditions for the circulation of the virus and, therefore, the appearance of autochthonous cases of dengue during certain months of the year. These include the presence of a competent vector (*Ae. albopictus*), a substantial flow of travellers from areas with active transmission of dengue that can introduce the virus and adequate climatic conditions to maintain the transmission of the virus once introduced. The most probable period in which autochthonous cases may appear is from May to October–November because of greater vector activity and density, although *Aedes* activity has been detected in some parts of Spain even in December. In Ibiza, the period of *Ae. albopictus* activity starts between April and May, with a peak of abundance between September and October. Therefore, the season of vector activity coincides with the maximum number of tourists visiting the island. According to official sources, in 2022, Ibiza hosted 3,407,433 tourists, with 12.7% coming from Germany [22].

The detection of several cases among tourists and the presence of a viraemia in a patient with a travel-associated infection in the same location suggest that the most likely transmission was through mosquito bites, but other possibilities cannot be totally excluded. Although the source case assured that they had not been bitten by mosquitoes and that they had spent the entire symptomatic period at home (in this case, the symptomatic period was coincident with viraemic period based on their arrival to the island), it is possible that the bites went unnoticed. Given this outbreak, the services in charge of vector control may need to reassess whether it is reasonable to base their risk assessment on the case's testimony.

Our investigation had some limitations. Confirmation through molecular techniques (a positive result by PCR or by antigen detection) could not be performed in the source case and Case 2 in this outbreak because of the lack of stored samples. Samples were also not available to perform further analysis like seroconversion or increase of the IgG titres in convalescent samples or neutralisation tests. Moreover, the possible cases (travel companions of Case 1 and Case 2) unfortunately were not tested at all, even when a strong epidemiological plausibility existed, given the timeframe of symptom onset and epidemiological parameters. 

Interestingly, the lack of detection of additional cases in Ibiza residents, especially during the temporal gap between the two German cases, suggests that some cases probably linked to this outbreak may have gone undetected; as a large proportion of dengue cases remain asymptomatic, patients with mild symptoms may not have sought medical attention and other possibly infected tourists may not have been tested for DENV in their home countries. The absence of autochthonous dengue risk in Spain probably led to a low perception of the disease of the practitioners. Therefore, if a case with compatible symptoms would present at a healthcare centre, it may be unlikely that a DENV test would be performed, both in Spain and in international tourists’ home countries. The special surveillance of dengue implemented in Ibiza in 2023 will be very useful to understand if there really are cases of dengue that go unnoticed among the local population. 

Health professionals have an important role in the surveillance, prevention and control of vector-borne diseases, in particular during periods of high abundance of the vector and high season of travellers. Given the scenario found in the Balearic Islands, i.e. presence of a competent mosquito vector yet still free of endemic dengue, we propose three key messages to health professionals. The first message is to encourage physicians to recommend that their patients with travel-associated dengue in their symptomatic phase protect themselves against mosquito bites. The second message for physicians attending an imported dengue case is that they notify public health professionals immediately so that they can carry out vector control measures around the case, if needed. Moreover, the Spanish National Prevention, Surveillance, and Control Vector-Borne Diseases Plan recommends that travellers protect themselves from mosquito bites for 2 weeks after returning from endemic countries. This measure is important to prevent transmission of the virus to mosquitos during the viraemic period including the pre-symptomatic phase and in asymptomatic cases [23]. The third key message would be for the services in charge of vector control to reassess the value of patients’ information on mosquito exposures in their risk assessment for the necessity of vector control around the case.

In Europe and other non-dengue-endemic countries, more emphasis needs to be placed on reliable laboratory testing or rapid confirmation in cases with travel history to areas where dengue vectors are established, e.g. in Southern Europe, during the virus transmission season. Also, available laboratory results should immediately be notified in full to aid in rapid confirmation. This cluster represents the second time that autochthonous dengue transmission on the Mediterranean coast has been detected via infected German tourists [24]. This outbreak demonstrates the usefulness of thorough and extensive surveillance of non-autochthonous diseases in travel returnees for both the country of residence as well as the countries where exposures took place.

It is very important to increase the awareness of detection of dengue cases in Spain and other European countries where *Ae. albopictus* is established – through educational plans targeting citizens and clinicians – to prevent and control autochthonous cases. As has happened in Ibiza, most of the breeding places for the tiger mosquito, i.e. containers where water accumulates, are found on private properties. Therefore, for the prevention of vectors, education of the population to recognise and avoid these breeding places is essential. Citizen involvement is essential to contribute to the control of *Ae. albopictus*. Through the platforms, citizens can report bites and send photos of mosquitoes, which can be helpful to public health authorities, to implement measures. Municipalities have also the responsibility of controlling mosquitoes in public areas, i.e. sewage. Periodic mosquito surveillance at the municipality level and quality control of the control measures taken, i.e. larval and/or adult control, are key tools to reduce the risk of transmission from imported cases.

## Conclusions

This outbreak description provides an example of how travellers returning from an endemic area and infected with dengue virus can cause a local outbreak in Europe, in areas where *Ae. albopictus* mosquitoes is established. The detection of travel associated dengue must be rapidly communicated to public health authorities in order to guide relevant prevention and control measures to avoid further transmission.

## Supplementary Data

307000 Supplement
