# Supplementary material for: Detection of dengue in German tourists returning from Ibiza, Spain, related to an autochthonous outbreak, August to October 2022
Source: Euro Surveill. 2024 Apr 4;29(14):2300296. doi: 10.2807/1560-7917.ES.2024.29.14.2300296 (PMC11004590; doi:10.2807/1560-7917.ES.2024.29.14.2300296)
Supplement: Supplement [file 23-00296_GARCIA_SAN_MIGUEL_Supplement.pdf]

## Supplementary Material

This supplementary material is hosted by Eurosurveillance as supporting information alongside the article [Detection of dengue in German tourists returning from Ibiza, Spain, related to an autochthonous outbreak, August to October 2022] on behalf of the authors who remain responsible for the accuracy and appropriateness of the content. The same standards for ethics, copyright, attributions and permissions as for the article apply. Eurosurveillance is not responsible for the maintenance of any links or email addresses provided therein.

**Figure S1**. Travel associated cases of dengue, 2015-2023, Balearic Islands

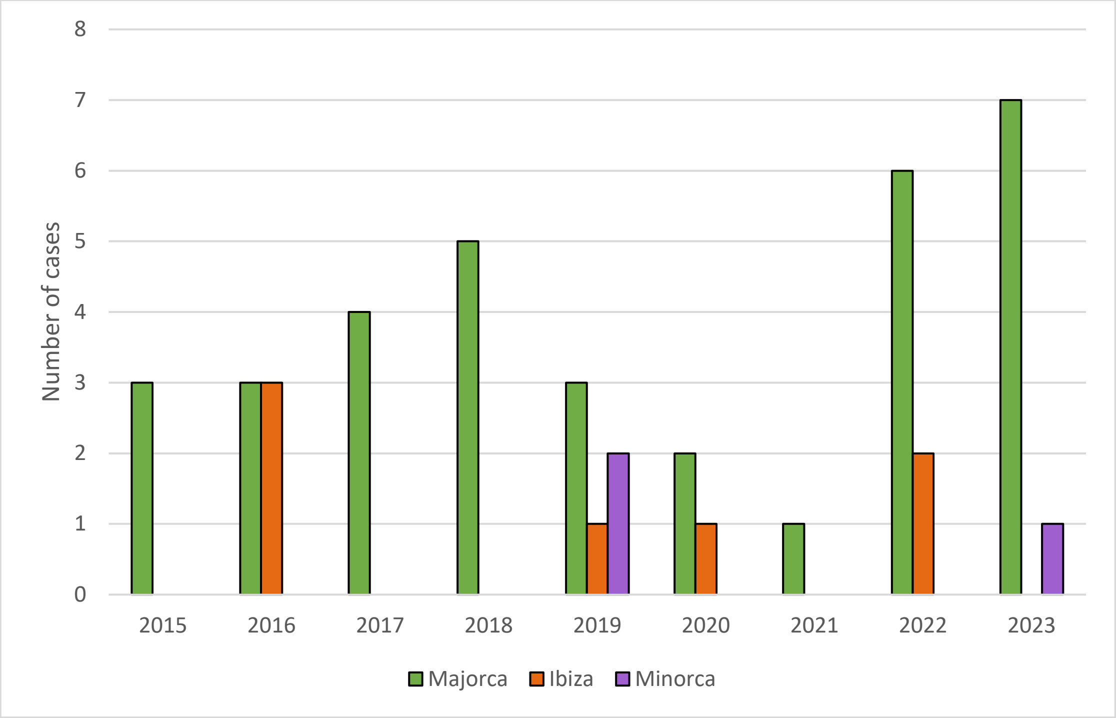


Source: Spanish National Surveillance Network (Red Nacional de Vigilancia Epidemiológica, RENAVE)

**Figure S2**. Surveillance of *Ae. albopictus* by municipality in Spain, 2009-2022

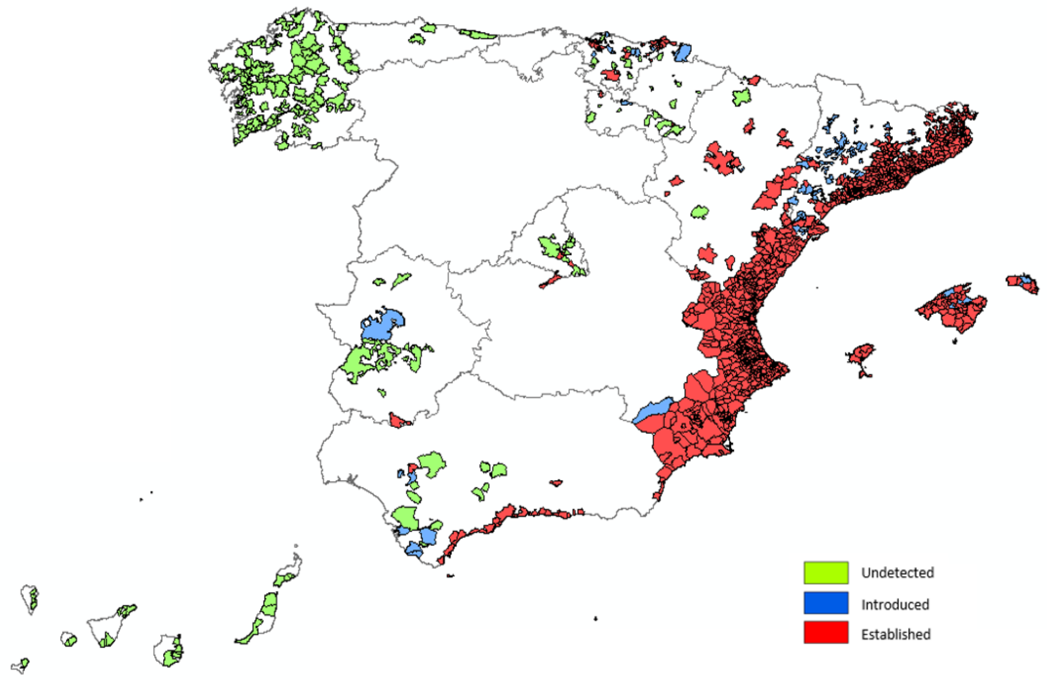


Introduced (blue): first and only detection in the last 3 years of entomological surveillance; Established (red): detected repeatedly for more than a year; Absent (green): entomological surveillance and always negative results or three consecutive years being negative after a positive result; No data (white): more than three years without entomological surveillance.

Source: Health Alerts and Emergencies Coordination Centre, Ministry of Health, Spain
